# Cervical mucus proteome in endometriosis

**DOI:** 10.1186/s12014-017-9142-4

**Published:** 2017-02-02

**Authors:** Giuseppe Grande, Federica Vincenzoni, Domenico Milardi, Giuseppina Pompa, Domenico Ricciardi, Erika Fruscella, Francesca Mancini, Alfredo Pontecorvi, Massimo Castagnola, Riccardo Marana

**Affiliations:** 1 International Scientific Institute “Paul VI”, L.go F. Vito 1, 00168 Rome, Italy; 2 Institute of Biochemistry and Clinical Biochemistry, Catholic University, L.go F. Vito 1, 00168 Rome, Italy; 3 Department of Obstetrics and Gynecology, Fondazione Policlinico Universitario A. Gemelli, L.go F. Vito 1, 00168 Rome, Italy; 4 Division of Endocrinology, Catholic University, L.go F. Vito 1, 00168 Rome, Italy

**Keywords:** Cervical mucus, Endometriosis, Non-invasive, Markers, Proteomic

## Abstract

**Background:**

Endometriosis is a chronic gynecological inflammatory disease characterized by the presence of functional endometrial glands and stroma outside of the uterine cavity. It affects 7–10% of women of reproductive age and up to 50% of women with infertility. The current gold standard for the diagnosis combines laparoscopic evaluation and biopsy of the visualized lesions. However, laparoscopy requires general anesthesia and developed surgical skills and it has a high procedural cost. In addition, it is associated with the risk, although rare, of potential intraoperative or postoperative complications. To date, several noninvasive biomarkers have been proposed; however, no definite diagnostic biomarker is yet available. The aim of this study was to characterize the CM proteome in patients with endometriosis using high resolution mass spectrometry—based proteomics, implemented by bioinformatic tools for quantitative analysis, in order to investigate the pathophysiological mechanisms of endometriosis.

**Methods:**

Cervical mucus samples were collected from patients affected by endometriosis and fertile controls. An aliquot of the soluble acidic fraction of each cervical mucus sample, corresponding to 0.5 mg of total protein, was left to digest with sequencing grade modified porcine trypsin. The peptides were analyzed by LC–MS/MS on a high resolution Orbitrap Elite mass spectrometer and data were evaluated using bioinformatic tools.

**Results:**

We aimed at the first total profiling of the cervical mucus proteome in endometriosis. From the list of identified proteins, we detected a number of differentially expressed proteins, including some functionally significant proteins. Six proteins were quantitatively increased in endometriosis, almost all being involved in the inflammatory pattern. Nine proteins were quantitatively reduced in endometriosis, including some proteins related with local innate immunity (CRISP-3 and Pglyrp1) and protection against oxidative stress (HSPB1). Fifteen proteins were not detected in endometriosis samples including certain proteins involved in antimicrobial activity (SLURP1 and KLK13) and related to seminal plasma liquefaction and male fertility (KLK13).

**Conclusions:**

This is the first application of high resolution mass spectrometry—based proteomics aimed in detecting an array of proteins in CM to be proposed for the noninvasive diagnosis of endometriosis. This chronic disease presents in CM an inflammatory protein pattern.

**Electronic supplementary material:**

The online version of this article (doi:10.1186/s12014-017-9142-4) contains supplementary material, which is available to authorized users.

## Background

Endometriosis is a chronic gynecological inflammatory disease characterized by the presence of functional endometrial glands and stroma outside of the uterine cavity [1] that affects 7–10% of women of reproductive age and up to 50% of women with infertility [2]. Many studies report lower fertility rates in women with endometriosis compared to patients free from this disease. Many mechanisms have been suggested to explain the decrease in fertility of these women, such as altered folliculogenesis [3], poor oocyte quality [4], luteal phase defects [5], sperm damage [6], embryo implantation failure [7], abnormal embryogenesis [8]. Studies reported that endometriosis could interfere with the receptivity of eutopic endometrium [9, 10] and it is associated with abnormal prostaglandin-E2 and cytokine production [11]. Yet, the underlying molecular mechanics that may participate in each of these events is still poorly understood.

Up to 60% of the women affected by endometriosis present characteristic symptoms such as dysmenorrhea, dyspareunia, dyschezia, dysuria and cyclic back pain, as well as cyclic and noncyclic lower abdominal pain [12]. However, recognition and conclusive diagnosis only occur on the average approximately 9 years following the beginning of the disease [13]. Factors responsible for such a delayed diagnosis include asymptomatic course  the of disease, nonspecific symptoms and inconclusive findings in non-invasive examinations. The current gold standard for the diagnosis of the disease combines laparoscopic evaluation and biopsy of the visualized lesions [14]. Although laparoscopy is a minimally invasive procedure, it requires general anesthesia and developed surgical skills, and it has a high procedural cost. In addition, laparoscopy is associated with the risk, although rare, of potential intraoperative or postoperative complications [15].

Early detection would allow individuals affected by the disease more options for treatment, i.e. making earlier surgical treatment more effective and assist in the search for factors involved in pathogenesis. Even though the urgent need for early non-invasive diagnosis was well recognized over a half-century ago [16], no significant contributions to endometriosis diagnosis have been made in this field.

Currently, no non-invasive imaging modalities that can be used to accurately diagnose endometriosis are available in clinical practice.

A recent Cochrane database systematic review [17] concluded that rectosigmoid endometriosis is the only site that could be accurately mapped by using trans-vaginal ultrasound (TVUS), trans-rectal ultrasound (TRUS), magnetic resonance imaging (MRI) or multi-detector computerized tomography enema (MDCT-e). Specifically for endometrioma, TVUS and MRI displayed sufficient accuracy to suggest their utility as a replacement test. However, the data were too scarce to permit meaningful conclusions. Instead of MRI, TVUS could be used clinically to identify additional anatomical sites of deep infiltrating endometriosis, thus facilitating preoperative planning.

To date none of the evaluated imaging modalities is able to detect overall pelvic endometriosis with enough accuracy to be suggested for replacing surgical diagnosis.

To date, several noninvasive biomarkers have been proposed as an adjunct in diagnostics. Cancer antigen 125 (CA 125) is the most widely used biomarker of endometriosis; however, despite the finding of higher CA 125 levels in some women with endometriosis, CA 125 may be a poor biomarker because it does not perform well in the detection of the disease [18]. Diagnostic markers have been searched in peritoneal [19], follicular [20], and endometrial fluid [21], in urine [22], and in blood cells [23] however, no definite diagnostic biomarker is yet available [17].

It is widely accepted that eutopic endometrium from endometriosis patients differ from eutopic endometrium from healthy women. Recent studies reported that matrix metalloproteinase-2 (MMP-2), MMP-9, tissue inhibitor of matrix metalloproteinases (TIMP-1) and transforming growth factor-β2 (TGF-β2) express a tendency to higher gene expression in the eutopic endometrium of women with endometriosis [24]. Euctopic endometrioum from endometriosis patients expresses moreover different mi-RNA profiles [25] and an aberrant DNA methylome [26].

In addition, because implants of endometriosis in the pelvic cavity release substances in the peritoneal fluid that bathe the ovary and enter the uterine cavity through the oviducts, endometriotic markers may influence the entire reproductive system, including the cervical mucus (CM). No studies have been performed up to now to verify the possibility of identifying non-invasive biomarkers of endometriosis in CM.

The development of high-throughput technologies, such as proteomics, has led to an early identification of the diseases by comparing the protein composition in diseased and normal tissues [27]. In a previous study we confirmed that CM is a source of protein biomarkes and identified the constitutive protein composition of CM of fertile women and the changes in the CM proteome throughout the menstrual cycle [28].

The aim of this study was to characterize the pattern of proteins involved in the pathophysiology of endometriosis. This aim could be important to furtherly focus which of these proteins could represent putative markers of endometriosis in CM.

## Methods

### Study design

Ten 30–40 year old infertile women, with ovarian endometriotic cysts were enrolled. Ten fertile women, with no history of fertility problems and who had a term delivery within 1 year before the study served as control group.

The inclusion criterion was a maximum age of 40 years for both groups. A specific inclusion criterion for the patients with endometriosis was that the endometriosis diagnosis was obtained by videolaparoscopy and confirmed by histological exam of the endometiotic cyst.

The exclusion criteria for both groups were as follows: patients with a history of polycystic ovary syndrome, cancer, premature ovarian failure, or other gynecologic factors leading to infertility that could affect folliculogenesis.

Cervical and vaginal swabs were obtained before sample collection to exclude cervical and vaginal infection. PAP tests and colposcopy were also performed before sample collection.

### CM collection and sample preparation

Cervical mucus samples (n = 1 per patient) were obtained by gentle aspiration from the cervical canal using a catheter for intrauterine insemination (Gynetics Medical Products, Achel, Belgium). Samples were collected during the ovulation phase of the menstrual cycle. Ovulation was assessed by transvaginal sonography and confirmed by the measurement of midluteal serum progesterone levels.

The cervical mucus was collected in plastic tubes and mixed 1:1 (v/v) with aqueous trifluoroacetic acid solution (0.2% v/v) and centrifuged at 9200*g* for 10 min. The soluble acidic fraction was stored at −80 °C until analysis. An aliquot of the soluble acidic fraction of each CM sample, corresponding to 40 µg of total protein (as measured by the Bradford assay), was mixed with 1 M ammonium bicarbonate pH 8.0 and reduced with 200 mM dithiothreitol (DTT 10 mM final, Sigma) for 5 min at 100 °C and 15 min at 50 °C, and then alkylated with 200 mM iodoacetamide (IAA 55 mM final, Sigma) in the dark at room temperature for 60 min. The samples were left to digest overnight at 37 °C by adding 100 mM ammonium bicarbonate (pH 8) with sequencing grade modified porcine trypsin (1:50, trypsin:protein concentration, Pierce Biotechnology). To stop the digestion, the samples were acidified with aqueous trifluoroacetic acid solution (0.2% v/v) and immediately frozen and lyophilized.

### Proteomic analysis

The samples were resuspended in 40 μl of aqueous formic acid solution (0.1% v/v) and equal protein quantity (8 μg) of each sample was analyzed using an Ultimate 3000 RSLCnano equipped with an FLM-3000-Flow manager module, and coupled using an Orbitrap Elite mass spectrometer (ThermoFisher, San Jose, CA). Separation experiments were performed using a Zorbax 300SB-C18 column (3.5 mm particle diameter; column dimension 1 mm i.d. 15 cm) (Agilent Technologies, Santa Clara, CA) using the following eluents: (A) 0.1% (v/v) aqueous formic acid and (B) acetonitrile:water 80:20 with 0.1% (v/v) aqueous formic acid. The applied gradient was linear from 0 to 55% of solvent B in 60 min, at a flow rate of 50 μl/min. The Elite-Orbitrap mass spectrometer was operated in data-dependent mode in which each full MS scan (60 000 resolving power) was followed by MS/MS scans where the five most intense multiple-charged ions were dynamically selected and fragmented by collision-induced dissociation (CID) at a normalized collision energy of 35%. Samples were analyzed individually; proteomic analysis was performed at the same time for all samples, while data analysis was subsequently performed.

### Data analysis

Tandem mass spectra were analysed using the Thermo Proteome Discoverer 1.4 software, and the SEQUEST cluster (University of Washington, Seattle, WA, licensed by Thermo Electron Corp) as the search engine against UniProtKb/Swiss-Prot protein knowledgebase release 2015-10: 20196 Homo Sapiens protein database.

In order to obtain a reliable identification of the peptides, the following filters were used: high value peptide confidence and false discovery rate (FDR) of 5%.

Data were searched for three missed cleavages, fixed carbamidomethylation of cysteines and the oxidation of methionines as variable modification.

The label-free quantification of common proteins was performed via Precursor Ions Area Detector Node during the bioinformatic analysis using Proteome Discoverer software. This quantification method was used to obtain an idea of the relative quantities of all peptides in a sample. The Proteome Discoverer application calculates peptide areas during processing, using them to automatically calculate protein areas for the proteins in the report. It calculates the area of any given protein as the average of the three most abundant distinct peptides identified in the protein.

Results are reported as average ± standard deviation. Statistical analysis was carried out with SPSS v17.0 (IBM Corp., Armonk,NY, USA). All data were first analyzed for normality of distribution using the Kolmogorov–Smirnov test of Normality. Since data were not normally distributed, the appropriate non-parametric (Mann–Whitney) test was used to assess significance of the differences between groups. p value <0.05 was considered as significant.

For the aim of this study, we considered the panel of proteins exclusively identified in the endometriosis group and in the control group. Moreover we evaluated the proteins significantly increased or reduced according to abundance index in the endometriosis versus the control group.

## Results

Protein identification led to the characterization of a range of 44–140 different proteins per sample in the endometriosis group and 91–126 proteins in the control group.

All the proteins we identified in the endometriosis group have been detected in the control group.

**Fig. 1:**
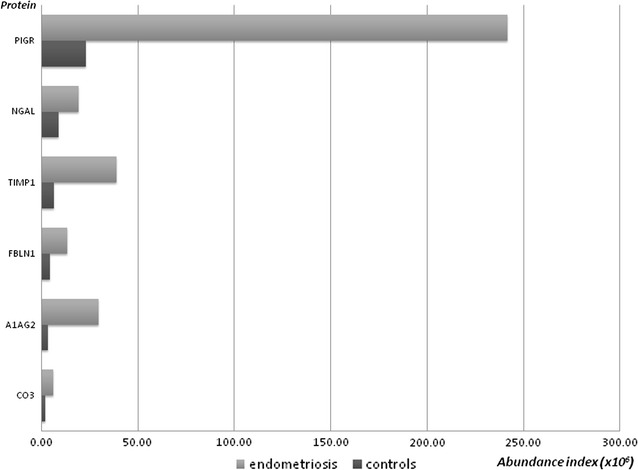
Protein abundance in endometriosis and controls—proteins increased in patients with endometriosis

Six proteins were quantitatively increased in endometriosis, as reported in Fig. 1 and almost all are involved in the inflammatory pattern.

**Fig. 2:**
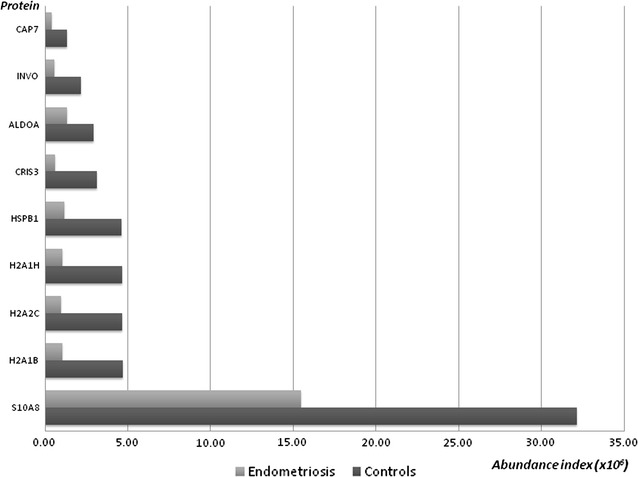
Protein abundance in endometriosis and controls—proteins decreased in patients with endometriosis

Nine proteins were quantitatively reduced in endometriosis, as reported in Fig. 2, including some proteins related with local innate immunity (CRISP-3 and Pglyrp1) and protection against oxidative stress (HSPB1).

Additional file 1: Table S1 reports the identified proteins in the two groups.

Table 1 reports the mean values of the abundance index for each protein in the two groups, the number of samples in which the proteins have been detected in patients and controls and if the protein was or not previously reported as a marker of endometriosis.

**Table 1:** Proteins reduced or increased in endometriosis versus controls

Accession	Description	Gene	No. of samples in which the protein was detected in control group	No. of samples in which the protein was detected in control group	Abundance index in controls (average ± SD) (×10^6^)	Abundance index in patients (average ± SD) (×10^6^)	Previously associated with endometriosis
P01833	Polymeric immunoglobulin receptor	PIGR	10	10	23.18 ± 14.63	241.86 ± 135.40	No
P19652	Alpha-1-acid glycoprotein 2	A1AG2	8	10	3.37 ± 3.07	29.62 ± 20.06	Yes, in peritoneal fluid [71]
P23142	Fibulin-1	FBLN1	10	10	4.52 ± 5.09	13.44 ± 5.61	Yes, in endometrium [72]
P01033	Metalloproteinase inhibitor 1	TIMP1	10	10	6.32 ± 5.64	38.98 ± 27.67	Yes, in endometrium [24]
P80188	Neutrophil gelatinase-associated lipocalin	NGAL	10	10	8.72 ± 5.14	19.35 ± 9.40	Yes, in urine [50]
P01024	Complement C3	CO3	8	10	2.03 ± 3.56	5.93 ± 2.37	Yes, in peripheral blood [73]
P54108	Cysteine-rich secretory protein 3	CRIS3	10	6	3.11 ± 1.62	0.58 ± 0.11	No
P04908	Histone H2A type 1-B/E	H2A1B	10	6	4.68 ± 3.22	1.04 ± 1.08	No
P07476	Involucrin	INVO	8	6	2.18 ± 1.56	0.55 ± 0.88	No
Q16777	Histone H2A type 2-C	H2A2C	10	4	4.66 ± 3.80	0.97 ± 1.84	No
Q96KK5	Histone H2A type 1-H	H2A1H	10	4	4.66 ± 3.58	1.03 ± 0.77	No
P04792	Heat shock protein beta-1	HSPB1	10	4	4.61 ± 0.97	1.15 ± 1.84	No
P20160	Azurocidin	CAP7	8	4	1.31 ± 0.97	0.38 ± 0.06	No
P05109	Protein S100-A8	S10A8	10	10	32.12 ± 17.23	15.45 ± 12.06	No
P04075	Fructose-bisphosphate aldolase A	ALDOA	10	4	2.94 ± 1.21	1.32 ± 0.14	No

Fifteen proteins were not detected in endometriosis samples compared to controls (Table 2) including some proteins involved in antimicrobial activity (SLURP1 and KLK13) or previously reported as related to seminal plasma liquefaction and male fertility (KLK13).

**Table 2:** Proteins not detected in CM of patients with endometriosis

Accession	Description	Σ coverage	Σ# proteins	Σ# unique peptides	Σ# peptides	No. of samples the protein was detected in the control group	MW (kDa)	X score (mean)	Calc. pI
P01040	Cystatin-A	70.41	1	5	5	8	11.0	15.01	5.50
P55000	Secreted Ly-6/uPAR-related protein 1	52.43	1	2	2	8	11.2	12.88	5.33
Q9UKR3	Kallikrein-13	36.10	1	6	6	10	30.6	18.10	8.46
O75594	Peptidoglycan recognition protein 1	26.53	1	3	3	8	21.7	11.65	8.59
P02671	Fibrinogen alpha chain	24.71	1	12	12	8	94.9	26.72	6.01
P10153	Non-secretory ribonuclease	22.98	1	4	4	8	18.3	11.93	8.73
O95274	Ly6/PLAUR domain-containing protein 3	21.97	1	4	4	10	35.9	25.57	7.75
P12724	Eosinophil cationic protein	20.00	1	3	3	8	18.4	13.78	10.02
P52566	Rho GDP-dissociation inhibitor 2	19.90	1	3	3	8	23.0	9.13	5.21
O75015	Low affinity immunoglobulin gamma Fc region receptor III-B	19.31	1	3	3	8	26.2	11.20	6.71
P00338	L-lactate dehydrogenase A chain	14.76	1	3	3	8	36.7	9.94	8.27
P32926	Desmoglein-3	10.01	1	7	7	8	107.5	43.29	5.00
Q02487	Desmocollin-2	8.88	1	4	4	8	99.9	29.56	5.34
P07477	Trypsin-1	7.29	1	2	2	9	26.5	7.55	6.51
P0C0L4	Complement C4-A	1.95	2	2	2	9	192.7	9.55	7.08

## Discussion

In the postgenomic era, proteomic technology has rapidly developed and it has become a powerful tool in the research of human physiology, in particular in biological fluids, characterizing the comprehensive proteomic composition and identifying potential novel biomarkers for diagnosis, prognosis and therapy in different clinical aspects, including reproduction [29]. Cervical mucus is a biofluid, representing a source of putative biomarkers for female genital tract diseases. The proteomic composition of cervical mucus is less complex when compared to plasma and urine, but few studies investigated cervical mucus composition in physiological conditions [28, 30] and no studies have been reported up to now concerning the identification of CM biomarkers for genital tract diseases.

We studied for the first time CM protein composition with the aim of identifying some proteins involved in the pathophysiology of endometriosis. The integration of high-resolution MS proteomic techniques and bioinformatic analysis permitted the identification of a pattern of proteins which could represent a molecular signature of endometriosis.

### Overexpressed proteins

Six proteins resulted overexpressed in endometriosis: 4 out to these 6 proteins are related with inflammation, including polymeric immunoglobulin receptor (pIgR), Alpha-1-acid glycoprotein 2, Metalloproteinase inhibitor 1 and Neutrophil gelatinase-associated lipocalin. Inflammatory processes have a crucial role in the pathogenesis of endometriosis [31] as suggested by the abnormal levels of immune system cells, including macrophages [32], dendritic cells [33] and natural killer cells [34], within the female reproductive tract reported in patients with endometriosis. These cells are unable to completely eliminate ectopic endometrial cells. Moreover, immune system cells were found to be dysfunctional in endometriosis [34, 35]. Complicated reactions may occur due to endometriosis-induced secretion of cytokines [36], chemokines [37], nitric oxide [38], immunoglobulins [39], and immune cells [32–35]. Triggered immune reactions signify the host recognition of infectious agents, but, if pathogens are not swiftly recognized, immune reactions necessary to fight infections do not occur. Previous data reported an higher prevalence of endometritis in women with endometriosis [40], which may represent an adjunctive mechanisms to explain the decrease of fertility in these women.

The overexpressed proteins verified in this study confirm the presence of inflammatory protein pattern in CM of patients with endometriosis, even if vaginal and cervical swabs excluded vaginal and cervical infection in all patients.

In particular, Polimeric Immunoglobulin Receptor (pIgR) plays an important role in mucosal immune systems, since it binds and transports IgA in different mucosal tissues [41]. Previous data reported that IgA and IgG are differentially associated with the types of mucus of the female reproductive tract, since both IgA and IgG are stably associated with cervical mucus, but only IgG is associated with cervicovaginal mucus [42]. Antibodies can bind tightly to mucus, where they play a significant role in the fortification of the mucus barriers of the female reproductive tract.

Orosomucoid 2 (ORM2), also known as alpha 1 acid glycoprotein 2, is a type of acute-phase protein considered an anti-inflammatory and immunomodulatory factor due to its anti-neutrophil and anti-complement activity [43].

Tissue inhibitors of metalloproteinases (TIMP) are the inhibitors of matrix metalloproteinases. Expression of matrix metalloproteinases (MMPs) and their inhibitors (tissue inhibitors of metalloproteinases, TIMPs) is reportedly involved in the pathogenesis and pathophysiology of endometriosis. The MMPs and TIMPs are synthesized and secreted by both eutopic and ectopic endometrium in both the human and the rat [44, 45]. TIMP1 represents at least 10–15% of the proteins secreted into the peritoneal cavity by both rat implants and human endometriotic lesions [46]. Previous data reported that an excessive TIMP1 secretion in endometriosis was deleterious to ovulation and embryo development [47]. There are some literature reports about the expression of TIMP in CM, linking the expression of metalloproteinases-TIMP systems in the cervical mucus plug during pregnancy with the proteolytic activity in connection with term and pre-term birth [48]. We previously reported that TIMP-1 is a marker of pre-ovulatory CM in fertile patients that might be associated with the inhibition of proteolitic activity, which leads to the liquefaction of CM in the ovulatory phase [28]. In patients with endometriosis, the higher expression of TIMP-1 during ovulatory phase might contribute in inhibiting the proteolitic activity and the liquefaction of CM, which is essential for CM permeability to spermatozoa.

Neutrophil gelatinase-associated lipocalin (NGAL) was previously identified by a proteomic approach as a marker of neutrophil activity in cervical–vaginal fluid [49]. NGAL has been previously reported to be elevated in the urine of patients with endometriosis [50].

In conclusion, the proteic pattern of abacterial cervical inflammation associated with endometriosis might both reflect the dysfunctional immune pattern of endometriosis and represent a link with endometriosis-associated infertility.

### Down-expressed proteins in endometriosis

Nine proteins were reduced in endometriosis, including Azurocidin 1, an important inflammatory mediator [51], cysteine-rich secretory protein 3 (CRISP-3) and Heat shock protein beta-1 (HSPB1).

CRISP-3 is present in exocrine secretions and in secretory granules of neutrophilic granulocytes and is believed to play a role in local innate immunity, which is depressed in patients with endometriosis [52]. Furthermore CRISP proteins have been reported to exert a role in sperm-oocyte chemotaxis both in frogs and mouse [53].

We observed a reduction of the antioxidant protein HSPB1. It is a small heat shock protein involved in many cellular processes, which protects cells against oxidative stress [54]. Endometriosis has been previously reported to be linked with oxidative stress, altering follicular microenvironment and oocyte quality [55, 56]. Further studies will clarify if endometriosis is associated with oxidative stress pattern in cervical mucus and its correlation with female infertility.

### Proteins not detected in endometriosis samples

Fifteen proteins were not detected in endometriosis samples, including some proteins involved in antimicrobial activity and possibly associated with fertility.

Human tissue kallikreins are members of a large multigene family of 15 serine proteases. The human kallikrein 13 gene (KLK13) codes for a trypsin-like, secreted serine protease (hK13). It was reported to be expressed in various tissues [57] and found at high concentrations in secreted biological fluids such as seminal plasma, amniotic fluid, milk of lactating women, follicular fluid, and cerebrospinal fluid [58], but up to now not reported as expressed in cervical mucus. Previous studies reported that kallikreins are important in the control of sperm liquefaction and sperm motility [59]. Further studies are needed to clarify the functional role of hK13 in cervical mucus and the molecular significance of its absence in endometriosis. We may speculate that the absence KLK13 in endometriosis could have a role in infertility.

We observed the absence of secreted Ly6/urokinase-type plasminogen activator receptor-related protein (SLURP) 1 and 3 proteins. SLURP1 belongs to the Ly6/uPAR superfamily of proteins that participate in signal transduction, immune activation, and cell adhesion [60]. It is expressed in a variety of cells including immune cells [61], bronchial epithelial cells [62], primary sensory neurons [63], skin, gums, stomach, trachea and esophagus [64], oral keratinocytes [65], cornea [66] and exocervix [64]. In corneal tissue it was reported that SLURP1 is a soluble scavenger of urokinase-type plasminogen activator (uPAR) and of its soluble form (suPAR) [67]. Numerous studies have shown that systemic soluble urokinase plasminogen activator receptor (suPAR) levels increase in various inflammatory diseases. In recent years it has been considered a reliable diagnostic and prognostic marker of systemic inflammation. uPAR and its ligand are involved in numerous physiological and pathological pathways, which include the plasminogen activating pathway, regulation of pericellular proteolysis, modulation of cell adhesion, migration, and proliferation through interactions with proteins present in the extracellular matrix [68]. In a previous study we demonstrated that suPAR might represent an inflammatory marker in reproduction for the male accessory glands [69]. In cervical mucus of patients with endometriosis we reported the absence of the scavenger proteins SLURP-1 and SLURP-3. We may speculate that endometriosis might cause an increase in uPAR activation and suPAR production, which are associated with an inflammatory pattern. Further studies are needed to confirm these data.

In CM of patients with endometriosis we reported the differential expression of Peptidoglycan recognition protein 1 (Pglyrp1). Pglyrps are a family of pattern-recognition proteins that mediate innate immunity to bacterial pathogens via binding peptidoglycan moieties [70]. Previous literature data confirmed that subclinical uterine infection and endometritis are more frequent in women with endometriosis versus controls [40]. The absence of Pglyrp1 might represent a molecular mechanism involved in the higher prevalence of infections in patients with endometriosis.

## Conclusions

We identified in the CM of patients affected by endometriosis some proteins which are mainly related to the pathogenesis of inflammation and the induction of a dysfunctional immune system. These data are consistent with previous reports about endometriosis as a chronic inflammatory disease.

Despite the limitations of the study, which was performed using fertile women and not on patients with endometriosis-like symtoms, without endometriosis at laparoscopic and histological examination, as control group, this is the first application of high resolution mass spectrometry—based proteomics aimed to reveal an array of proteins in CM which might represent a molecular signature of endometriosis. Our data confirm at the same time both the inflammatory pattern and the impairment of the natural immune system in the CM of patients affected by endometriosis.

## Additional file

**Additional file 1: Table S1.** Identified proteins in CM in the group of controls and in patients affected by endometriosis.
