# Supplementary material for: Cervical mucus proteome in endometriosis
Source: Clin Proteomics. 2017 Feb 2;14:7. doi: 10.1186/s12014-017-9142-4 (PMC5290661; doi:10.1186/s12014-017-9142-4)
Supplement: Supplementary file 1 — Additional file 1: Table S1. Identified proteins in CM in the group of controls and in patients affected by endometriosis. [file 12014_2017_9142_MOESM1_ESM.docx]

**Table S1**: Identified proteins in CM in the group of controls and in patients affected by endometriosis

| Accession | Description | Gene | Identified in Controls | Identified in Patients |
| --- | --- | --- | --- | --- |
| A8K2U0 | Alpha-2-macroglobulin-like protein 1 | A2ML1 | X | X |
| B3EWG3 | Protein FAM25A | FM25A | X | X |
| O00391 | Sulfhydryl oxidase 1 | QSOX1 | X | X |
| O00560 | Syntenin-1 | SDCB1 | X | X |
| O00592 | Podocalyxin | PODXL | X | X |
| O14745 | Na(+)/H(+) exchange regulatory cofactor NHE-RF1 | NHRF1 | X | X |
| O15143 | Actin-related protein 2/3 complex subunit 1B | ARC1B | X | X |
| O43240 | Kallikrein-10 | KLK10 | X | X |
| O43490 | Prominin-1 | PROM1 | X | X |
| O43866 | CD5 antigen-like | CD5L | X | X |
| O60911 | Cathepsin L2 | CATL2 | X | X |
| O75015 | Low affinity immunoglobulin gamma Fc region receptor III-B | FCG3B | X |  |
| O75594 | Peptidoglycan recognition protein 1 | PGRP1 | X |  |
| O75882 | Attractin | ATRN | X | X |
| O95171 | Sciellin | SCEL | X | X |
| O95274 | Ly6/PLAUR domain-containing protein 3 | LYPD3 | X |  |
| O95969 | Secretoglobin family 1D member 2 | SG1D2 | X | X |
| O95994 | Anterior gradient protein 2 homolog | AGR2 | X | X |
| P00338 | L-lactate dehydrogenase A chain | LDHA | X |  |
| P00441 | Superoxide dismutase [Cu-Zn] | SODC | X | X |
| P00450 | Ceruloplasmin | CERU | X | X |
| P00558 | Phosphoglycerate kinase 1 | PGK1 | X | X |
| P00738 | Haptoglobin | HPT | X | X |
| P00746 | Complement factor D | CFAD | X | X |
| P00747 | Plasminogen | PLMN | X | X |
| P00751 | Complement factor B | CFAB | X | X |
| P00915 | Carbonic anhydrase 1 | CAH1 | X | X |
| P00918 | Carbonic anhydrase 2 | CAH2 | X | X |
| P01008 | Antithrombin-III | ANT3 | X | X |
| P01009 | Alpha-1-antitrypsin | A1AT | X | X |
| P01011 | Alpha-1-antichymotrypsin | AACT | X | X |
| P01023 | Alpha-2-macroglobulin | A2MG | X | X |
| P01024 | Complement C3 | CO3 | X | X |
| P01033 | Metalloproteinase inhibitor 1 | TIMP1 | X | X |
| P01034 | Cystatin-C | CYTC | X | X |
| P01037 | Cystatin-SN | CYTN | X | X |
| P01040 | Cystatin-A | CYTA | X |  |
| P01042 | Kininogen-1 | KNG1 | X | X |
| P01591 | Immunoglobulin J chain | IGJ | X | X |
| P01600 | Ig kappa chain V-I region Hau | KV108 | X | X |
| P01617 | Ig kappa chain V-II region TEW | KV204 | X | X |
| P01620 | Ig kappa chain V-III region SIE | KV302 | X | X |
| P01625 | Ig kappa chain V-IV region Len | KV402 | X | X |
| P01833 | Polymeric immunoglobulin receptor | PIGR | X | X |
| P01834 | Ig kappa chain C region | IGKC | X | X |
| P01857 | Ig gamma-1 chain C region | IGHG1 | X | X |
| P01859 | Ig gamma-2 chain C region | IGHG2 | X | X |
| P01860 | Ig gamma-3 chain C region | IGHG3 | X | X |
| P01861 | Ig gamma-4 chain C region | IGHG4 | X | X |
| P01871 | Ig mu chain C region | IGHM | X | X |
| P01876 | Ig alpha-1 chain C region | IGHA1 | X | X |
| P01877 | Ig alpha-2 chain C region | IGHA2 | X | X |
| P02533 | Keratin, type I 14 | K1C14 | X | X |
| P02538 | Keratin, type II 6A | K2C6A | X | X |
| P02647 | Apolipoprotein A-I | APOA1 | X | X |
| P02652 | Apolipoprotein A-II | APOA2 | X | X |
| P02671 | Fibrinogen alpha chain | FIBA | X |  |
| P02675 | Fibrinogen beta chain | FIBB | X | X |
| P02679 | Fibrinogen gamma chain | FIBG | X | X |
| P02749 | Beta-2-glycoprotein 1 | APOH | X | X |
| P02750 | Leucine-rich alpha-2-glycoprotein | A2GL | X | X |
| P02760 | Protein AMBP | AMBP | X | X |
| P02763 | Alpha-1-acid glycoprotein 1 | A1AG1 | X | X |
| P02765 | Alpha-2-HS-glycoprotein | FETUA | X | X |
| P02766 | Transthyretin | TTHY | X | X |
| P02768 | Serum albumin _ | ALBU | X | X |
| P02774 | Vitamin D-binding protein | VTDB | X | X |
| P02787 | Serotransferrin | TRFE | X | X |
| P02788 | Lactotransferrin | TRFL | X | X |
| P02790 | Hemopexin | HEMO | X | X |
| P03973 | Antileukoproteinase | SLPI | X | X |
| P04040 | Catalase | CATA | X | X |
| P04075 | Fructose-bisphosphate aldolase A | ALDOA | X | X |
| P04080 | Cystatin-B | CYTB | X | X |
| P04083 | Annexin A1 | ANXA1 | X | X |
| P04217 | Alpha-1B-glycoprotein | A1BG | X | X |
| P04406 | Glyceraldehyde-3-phosphate dehydrogenase | G3P | X | X |
| P04745 | Alpha-amylase 1 | AMY1 | x | x |
| P04792 | Heat shock protein beta-1 | HSPB1 | X | X |
| P04908 | Histone H2A type 1-B/E | H2A1B | X | X |
| P05090 | Apolipoprotein D | APOD | X | X |
| P05109 | Protein S100-A8 | S10A8 | X | X |
| P05156 | Complement factor I | CFAI | X | X |
| P05164 | Myeloperoxidase | PERM | X | X |
| P06396 | Gelsolin | GELS | X | X |
| P06702 | Protein S100-A9 | S10A9 | X | X |
| P06727 | Apolipoprotein A-IV | APOA4 | X | X |
| P06744 | Glucose-6-phosphate isomerase | G6PI | X | X |
| P07339 | Cathepsin D | CATD | X | X |
| P07355 | Annexin A2 | ANXA2 | X | X |
| P07476 | Involucrin | INVO | X | X |
| P07477 | Trypsin-1 | TRY1 | X |  |
| P07602 | Prosaposin | SAP | X | X |
| P07737 | Profilin-1 | PROF1 | X | X |
| P07900 | Heat shock protein HSP 90-alpha | HS90A | X | X |
| P08123 | Collagen alpha-2(I) chain | CO1A2 | X | X |
| P08185 | Corticosteroid-binding globulin | CBG | X | X |
| P08246 | Neutrophil elastase | ELNE | X | X |
| P08311 | Cathepsin G | CATG | X | X |
| P08603 | Complement factor H | CFAH | X | X |
| P08697 | Alpha-2-antiplasmin | A2AP | X | X |
| P08727 | Keratin, type I 19 | K1C19 | X | X |
| P09466 | Glycodelin | PAEP | X | X |
| P09486 | SPARC | SPRC | X | X |
| P0C0L4 | Complement C4-A | CO4A | X |  |
| P0CG05 | Ig lambda-2 chain C regions | LAC2 | X | X |
| P0CG48 | Polyubiquitin-C | UBC | X | X |
| P0DMV8 | Heat shock 70 kDa protein 1A | HS71A | X | X |
| P10153 | Non-secretory ribonuclease | RNAS2 | X |  |
| P10412 | Histone H1.4 | H14 | X | X |
| P10599 | Thioredoxin | THIO | X | X |
| P10909 | Clusterin | CLUS | X | X |
| P12109 | Collagen alpha-1(VI) chain | CO6A1 | X | X |
| P12429 | Annexin A3 | ANXA3 | X | X |
| P12724 | Eosinophil cationic protein | ECP | X |  |
| P13645 | Keratin, type I 10 | K1C10 | X | X |
| P13646 | Keratin, type I 13 | K1C13 | X | X |
| P13646 | Keratin, type I 13 | K1C13 | X | X |
| P13647 | Keratin, type II cytoskeletal 5 | K2C5 | X | X |
| P13796 | Plastin-2 | PLSL | X | X |
| P14384 | Carboxypeptidase M | CBPM | X | X |
| P14618 | Pyruvate kinase PKM | KPYM | X | X |
| P14780 | Matrix metalloproteinase-9 | MMP9 | X | X |
| P15291 | Beta-1,4-galactosyltransferase 1 | B4GT1 | X | X |
| P15328 | Folate receptor alpha | FOLR1 | X | X |
| P15924 | Desmoplakin | DESP | X | X |
| P15941 | Mucin-1 | MUC1 | X | X |
| P17213 | Bactericidal permeability-increasing protein | BPIB1 | X | X |
| P18065 | Insulin-like growth factor-binding protein 2 | IBP2 | X | X |
| P18669 | Phosphoglycerate mutase 1 | PGAM1 | X | X |
| P19013 | Keratin, type II cytoskeletal 4 | K2C4 | X | X |
| P19440 | Gamma-glutamyltranspeptidase 1 | GGT1 | X | X |
| P19652 | Alpha-1-acid glycoprotein 2 | A1AG2 | X | X |
| P19957 | Elafin | ELAF | X | X |
| P20061 | Transcobalamin-1 | TCO1 | X | X |
| P20160 | Azurocidin | CAP7 | X | X |
| P22528 | Cornifin-B | SPR1B | X | X |
| P22531 | Small proline-rich protein 2E | SPR2E | X | X |
| P22692 | Insulin-like growth factor-binding protein 4 | IBP4 | X | X |
| P22894 | Neutrophil collagenase | MMP8 | X | X |
| P23142 | Fibulin-1 | FBLN1 | X | X |
| P23527 | Histone H2B type 1-O | H2B1O | X | X |
| P25311 | Zinc-alpha-2-glycoprotein | ZA2G | X | X |
| P29508 | Serpin B3 | SPB3 | X | X |
| P31151 | Protein S100-A7 | S10A7 | X | X |
| P31949 | Protein S100-A11 | S10AB | X | X |
| P32119 | Peroxiredoxin-2 | PRDX2 | X | X |
| P32320 | Cytidine deaminase CDD_HUMAN] | CDD | X | X |
| P32926 | Desmoglein-3 | DSG3 | X |  |
| P35321 | Cornifin-A [SPR1A_HUMAN] | SPR1A | X | X |
| P35325 | Small proline-rich protein 2B | SPR2B | X | X |
| P52209 | 6-phosphogluconate dehydrogenase, decarboxylating | 6PGD | X | X |
| P52566 | Rho GDP-dissociation inhibitor 2 | GDIR2 | X |  |
| P52943 | Cysteine-rich protein 2 | CRIP2 | X | X |
| P54108 | Cysteine-rich secretory protein 3 | CRIS3 | X | X |
| P55000 | Secreted Ly-6/uPAR-related protein 1 | SLUR1 | X |  |
| P58062 | Serine protease inhibitor Kazal-type 7 | ISK7 | X | X |
| P59666 | Neutrophil defensin 3 | DEF3 | X | X |
| P60022 | Beta-defensin 1 | DEFB1 | X | X |
| P60709 | Actin | ACTB | X | X |
| P61158 | Actin-related protein 3 | ARP3 | X | X |
| P61626 | Lysozyme C | LYSC | X | X |
| P61769 | Beta-2-microglobulin | B2MG | X | X |
| P62805 | Histone H4 | H4 | X | X |
| P68032 | Actin | ACTC | X | X |
| P68871 | Hemoglobin subunit beta HBB | HBB | X | X |
| P69905 | Hemoglobin subunit alpha | HBA | X | X |
| P80188 | Neutrophil gelatinase-associated lipocalin | NGAL | X | X |
| P80511 | Protein S100-A12 | S10AC | X | X |
| P80748 | Ig lambda chain V-III region | LV302 | X | X |
| P98088 | Mucin-5AC | MUC5A | X | X |
| Q01469 | Fatty acid-binding protein | FABP5 | X | X |
| Q02487 | Desmocollin-2 | DSC2 | X |  |
| Q03405 | Urokinase plasminogen activator surface receptor | UPAR | X | X |
| Q07654 | Trefoil factor 3 | TFF3 | X | X |
| Q08380 | Galectin-3-binding protein | LG3BP | X | X |
| Q12841 | Follistatin-related protein 1 | FSTL1 | X | X |
| Q12889 | Oviduct-specific glycoprotein | OVGP1 | X | X |
| Q14508 | WAP four-disulfide core domain protein 2 | WFCD2 | X | X |
| Q16610 | Extracellular matrix protein 1 | ECM1 | X | X |
| Q16777 | Histone H2A type 2-C | H2A2C | X | X |
| Q8TDL5 | BPI fold-containing family B member 1 | BPIB1 | X | X |
| Q8WXI7 | Mucin-16 | MUC16 | X | X |
| Q96KK5 | Histone H2A type 1-H | H2A1H | X | X |
| Q9BRK5 | 45 kDa calcium-binding protein | CAB45 | X | X |
| Q9HC84 | Mucin-5B | MUC5B | X | X |
| Q9HD89 | Resistin | RETN | X | X |
| Q9NQ38 | Serine protease inhibitor Kazal-type 5 | ISK5 | X | X |
| Q9UBC9 | Small proline-rich protein 3 | SPRR3 | X | X |
| Q9UBG3 | Cornulin | CRNN | X | X |
| Q9UBX7 | Kallikrein-11 | KLK11 | X | X |
| Q9UHD0 | Interleukin-19 | IL19 | X | X |
| Q9UKR3 | Kallikrein-13 | KLK13 | X |  |
| Q9Y6R7 | IgGFc-binding protein | FCGBP | X | X |
